# Salvianolic Acid A, as a Novel ETA Receptor Antagonist, Shows Inhibitory Effects on Tumor in Vitro

**DOI:** 10.3390/ijms17081244

**Published:** 2016-08-02

**Authors:** Qiao Zhang, Shifeng Wang, Yangyang Yu, Shengnan Sun, Yuxin Zhang, Yanling Zhang, Wei Yang, Shiyou Li, Yanjiang Qiao

**Affiliations:** 1 School of Chinese Materia Medica, Beijing University of Chinese Medicine, No. 6 Wangjingzhonghuan South Road, Chaoyang District, Beijing 100102, China; zhangqiao@bucm.edu.cn (Q.Z.); wangshifeng@bucm.edu.cn (S.W.); louisyang@bucm.edu.cn (Y.Y.); Zhangyuxinwjzy@163.com (Yu.Z.); collean_zhang@163.com (Ya.Z.); 2 Pharmacogenetics, HD Biosciences, Co., Ltd., 590 Ruiqing Road, Zhangjiang Hi-Tech Park East Campus, Pudong New Area, Shanghai 201201, China; pkssn12@gmail.com; 3 Technical Department, ACEA Biosciences Inc., No. 5 Sandunxiyuan Road, Hangzhou 310030, China; paddy.yang@aceabio.com.cn; 4 Beijing Institute of Genomics, Chinese Academy of Sciences, No. 1 Beichen West Road, Chaoyang District, Beijing 100101, China

**Keywords:** Salvianolic acid A, endothelin A receptor, anticancer, antagonist, cardiotoxicity

## Abstract

Endothelin-1 (ET-1) autocrine and paracrine signaling modulate cell proliferation of tumor cells by activating its receptors, endothelin A receptor (ET_A_R) and endothelin B receptor (ET_B_R). Dysregulation of ET_A_R activation promotes tumor development and progression. The potential of ET_A_R antagonists and the dual-ET_A_R and ET_B_R antagonists as therapeutic approaches are under preclinical and clinical studies. Salvianolic acid A (Sal A) is a hydrophilic polyphenolic derivative isolated from *Salvia miltiorrhiza* Bunge (Danshen), which has been reported as an anti-cancer and cardio-protective herbal medicine. In this study, we demonstrate that Sal A inhibits ET_A_R activation induced by ET-1 in both recombinant and endogenous ET_A_R expression cell lines. The IC_50_ values were determined as 5.7 µM in the HEK293/ET_A_R cell line and 3.14 µM in HeLa cells, respectively. Furthermore, our results showed that Sal A suppressed cell proliferation and extended the doubling times of multiple cancer cells, including HeLa, DU145, H1975, and A549 cell lines. In addition, Sal A inhibited proliferation of DU145 cell lines stimulated by exogenous ET-1 treatment. Moreover, the cytotoxicity and cardio-toxicity of Sal A were assessed in human umbilical vein endothelial cells (HUVEC) and Human-induced pluripotent stem cell-derived cardiomyocytes (hiPS-CMs), which proved that Sal A demonstrates no cytotoxicity or cardiotoxicity. Collectively, our findings indicate that Sal A is a novel anti-cancer candidate through targeting ET_A_R.

## 1. Introduction

Endothelin-1 (ET-1) is a small vasoactive peptide overexpressed in plasma and tissue samples from patients with various solid cancers [1]. ET-1 effects are mediated by two distinct G protein-coupled receptors (GPCR), endothelin A receptor (ET_A_R) and endothelin B receptor (ET_B_R). Endothelin receptors in cancer cells can be activated through either autocrine production of ligand or production of ligand from stromal cells that may be expressed physiologically, or in response to cancer cells in a paracrine loop [2,3]. In vitro, ET-1 production has been detected in a number of human cancer cell lines, from such colorectal, stomach, breast, and prostate [4]. Furthermore, exogenous ET-1 added to ovarian and prostate cancer cells stimulates proliferation [5,6].

Early studies in various tumor cells revealed that spontaneous growth was significantly inhibited by ET_A_R antagonists, such as Atrasentan (ABT-627) and Zibotentan (ZD4054), demonstrating that endogenous ET-1 acts as an autocrine modulator of cell proliferation through ET_A_R [7,8,9]. ET-1 provided a growth stimulus to colorectal cancer cells; the signal was also propagated via the transactivation of the epidermal growth factor receptor (EGFR) [10,11,12]. Moreover, preclinical studies using combined treatment of Zibotentan and the EGFR inhibitor gefitinib suppressed proliferation, invasion, and vascular endothelial growth factor (VEGF) production in ovarian cancer cells [13]. Therefore, the discovery of novel ET_A_R antagonists would be helpful for anti-tumor procession.

Natural ingredients derived from traditional Chinese medicines have shown obvious advantages in the therapy of certain diseases, such as multi-drug resistant cancer [14,15,16]. Thus, looking for candidates from traditional Chinese medicine provides a new path for the development of new drugs. Salvia polyphenols (Salvianolic acid A, B, C, D) are major active ingredients in *Salvia miltiorrhizae* Bunge (also termed as Danshen in China). Salvianolic acid A (Sal A) possesses multiple pharmacological activities, such as antiplatelet, anti-thrombosis, improvement of microcirculation, anti-inflammation, and antioxidant [17,18,19]. Furthermore, in recent years, it has been recognized that Sal A exerted effects on drug-resistant breast cancer cells [20,21]. Nonetheless, the potential target for Sal A on suppressing tumor cell proliferation remains to be illustrated.

Here we identified Sal A as a potential antagonist of ET_A_R via calcium mobilization assay. The anti-proliferative effect was evaluated by multiple cell lines with or without exogenous ET-1 by cell viability and real-time cell analysis assay. Furthermore, Sal A exhibited neither remarkable cytotoxicity in human umbilical vein endothelial cells (HUVEC) nor cardiotoxicity in human-induced pluripotent stem cell-derived cardiomyocytes (hiPS-CMs).

## 2. Results

### 2.1. HEK293/Endothelin A Receptor (ET_A_R) Cell Line Validation

**Figure 1:**
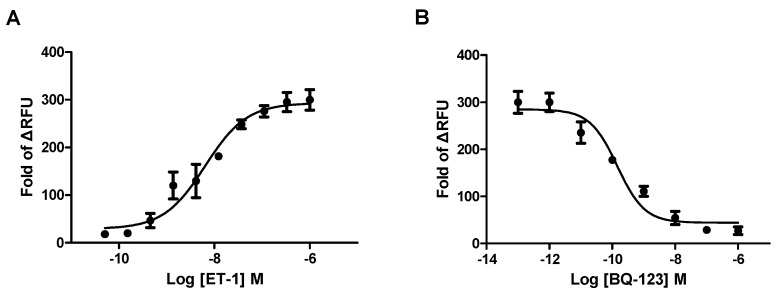
HEK293/endothelin A receptor (ET_A_R) cell line validation. (**A**) Agonist concentration–response curve of endothelin-1 (ET-1). The EC_50_ value of ET-1 was 4.78 nM; (**B**) Antagonist concentration–response curve of BQ-123 (evoked by 20 nM ET-1). The IC_50_ value of ET_A_R antagonist BQ-123 was 0.1 nM. Data were expressed as the fold change of max relative fluorescence units (RFU) and min RFU. Data were from three independent experiments.

The Gαq pathway is involved in ET_A_R activation, which increases intracellular Ca^2+^. Based on this characteristic, a cell-based calcium mobilization assay has been used to study the function of GPCR and Ca^2+^-permeable ion channels by measuring the changes of intracellular free Ca^2+^ levels. In our previous study, recombinant ET_A_R in HEK293 cells was developed according to standard procedures. The utility of the cell line was validated using ET_A_R agonist (ET-1) and antagonist (BQ-123). The EC_50_ value of ET_A_R agonist endothelin-1 (ET-1) was determined as 4.78 nM (shown in Figure 1), and the IC_50_ value of ET_A_R antagonist BQ-123 stimulated with 20 nM ET-1 (EC_90_ value) was determined as 0.1 nM, which were consistent with previously reported data [22].

### 2.2. ET_A_R Antagonist Primary Screening

**Figure 2:**
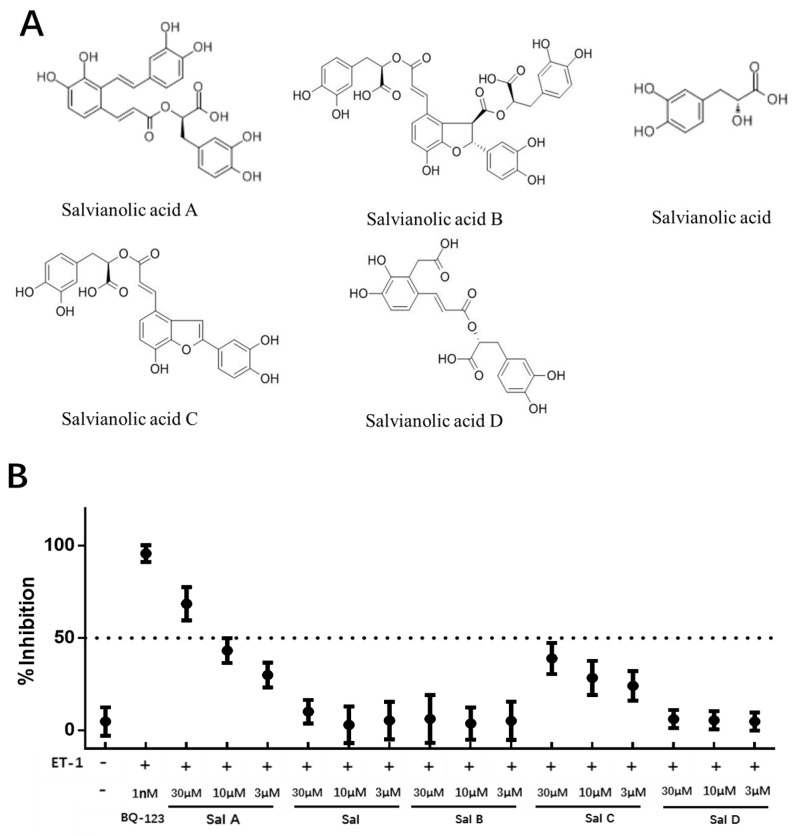
The primary evaluation of five compounds in HEK293/ET_A_R cell line. (**A**) Chemical formulas of Salvianolic acid A, Salvianolic acid B, Salvianolic acid C, Salvianolic acid D, Salvianolic acid; (**B**) Inhibitory responses of the five tested compounds in HEK293/ET_A_R cell line. The cells were pre-incubated with tested compounds for 10 min and then stimulated by 20 nM ET-1. Data (mean ± SD) were expressed as inhibition (%) from two independent experiments.

Salvianolic acid A (Sal A), Salvianolic acid B (Sal B), Salvianolic C (Sal C), Salvianolic acid D (Sal D), and Salvianolic acid (Sal); these five major active compounds of *Salvia miltiorrhizae* Bunge (also termed as Danshen in China) were tested in the primary screening, and their chemical formulas are shown in Figure 2A. These compounds were evaluated for inhibitory effect in HEK293/ET_A_R cells at various concentrations of 30, 10, and 3 µM using the cell-based calcium mobilization assay. BQ-123 (1 nM) was set as positive antagonist control and 0.25% Dimethyl sulfoxide (DMSO) was defined as vehicle control. The results suggested that Sal A should be an antagonist of ET_A_R, and the inhibitory effect of Sal A was concentration-dependent in the HEK293/ET_A_R cell line. In parallel, no obvious inhibitory effect was observed for the other tested compounds (Figure 2B).

### 2.3. Salvianolic Acid A (Sal A) Dose-Dependently Blocked Exogenous ET_A_R without Inducing Cytotoxicity in the HEK293/ET_A_R Cell Line

**Figure 3:**
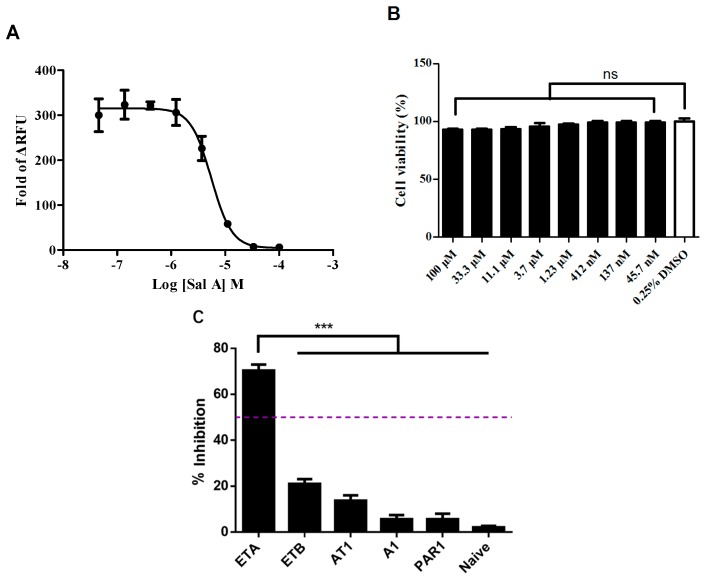
ET_A_R antagonist verification of Sal A. (**A**) Concentration-response curve in HEK293/ET_A_R cell line. The IC_50_ value of Sal A was 5.7 µM (evoked by 20 nM ET-1); (**B**) Cytotoxicity evaluation of Sal A in HEK293/ET_A_R cells. The cell viability was determined after 30 min compound treatment by using ATP CellTiter-Glo kit; (**C**) Selectivity assay of Sal A in five recombinant HEK293 cell lines. Purple dot-line: 50% inhibition. Data were expressed as means ± SD of three independent experiments. Statistical analysis by using one-way ANOVA with Tukey test. ns: no significance, *** *p* < 0.01. A1: adenosine A1 receptor; AT1: angiotensin II type 1 receptor; ETA: endothelin A; ETB: endothelin B; PAR1: proteinase-activated receptor 1.

To acquire the antagonist dose response, we tested the inhibitory effects of Sal A at different concentrations in the HEK293/ET_A_R cell line with 20 nM ET-1 stimulating. A dose-dependent trend of Sal A was observed with an IC_50_ value of 5.7 µM (Figure 3A). To omit a potential false positive, the cytotoxicity was tested in the HEK293/ET_A_R cell line by ATP CellTiter-Glo assay. In brief, Sal A was incubated in HEK293/ET_A_R cells for 30 min before luminescence signal measurement. Sal A did not show obvious cytotoxicity in the HEK293/ET_A_R cell line within 45.7 nM to 100 µM, compared with vehicle control (Figure 3B). The selectivity of Sal A was further assessed by calcium influx assay on 5 GPCRs. HEK293 cell lines stably expressing human ET_A_ receptors, ET_B_ receptors, adenosine A1 receptor (A1), angiotensin II type 1 receptor (AT1), and proteinase-activated receptor 1 (PAR1) were used. All cell lines were developed by standard procedures. Sal A was tested at a final concentration of 10 µM when cells were challenged with their selective agonists for antagonist identification. The selective antagonists of specific cell lines were used as positive control (Table S1). The HEK293 cell line was set up as the naïve group, and cells were challenged with 10 µM ATP as an agonist. The results showed that Sal A only interacted with the ET_A_ receptor in the screening panel, suggesting that Sal A is a selective ET_A_R antagonist (Figure 3C).

### 2.4. Sal A Inhibited Endogenously Expressed ET_A_R in HeLa Cells

**Figure 4:**
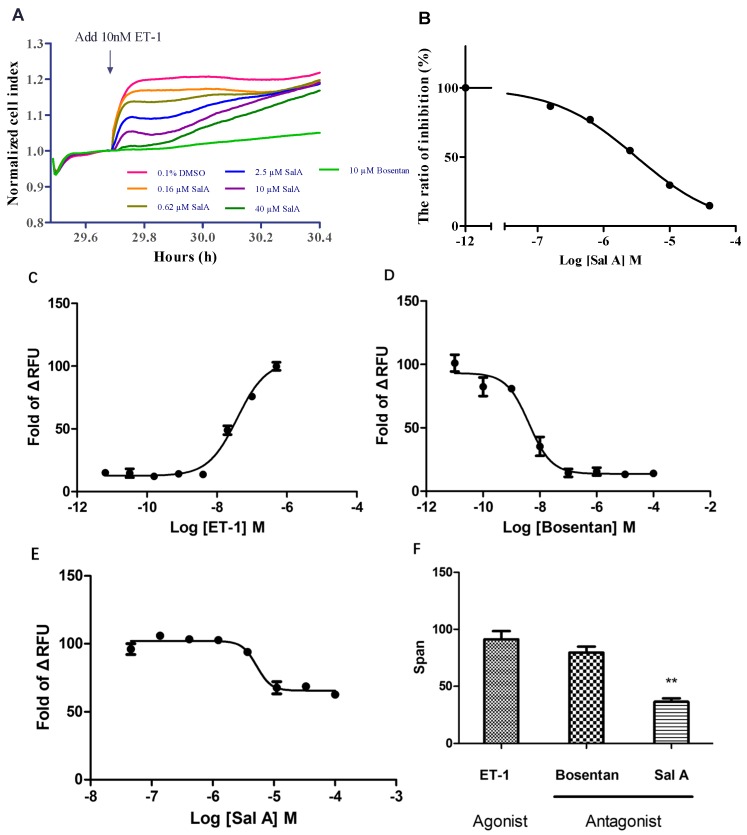
Effect of Sal A on (**A**,**B**) ET-1 stimulated cell index and (**C**–**F**) calcium influx in HeLa cells. (**A**) Time-dependent cell index (CI) curves are shown, dose concentration of Sal A and 10 µM Bosentan (positive control) were added at 29.4 h. After 15 min, 10 nM ET-1 was added. Black arrow: compound addition; (**B**) Concentration–response curves of Sal A calculated by (Max_CI_ − Min_CI_) during 29.7 to 30.4 h. The IC_50_ value of Sal A was 3.14 µM; (**C**) Concentration–response curve of ET-1 agonist in HeLa cells. The EC_50_ value was 39 nM; (**D**) Concentration–response curves of Bosentan in HeLa cells. The IC_50_ value was 4.3 nM; (**E**) Concentration–response curves of Sal A in HeLa cells. The IC_50_ value was 5.19 µM; (**F**) The comparison with response-span of three compounds. Data (**C**–**E**) were expressed as the fold change of max RFU and min RFU. Data are expressed as means ± SD of three independent experiments. Statistical analysis was conducted by using one-way ANOVA with Tukey test. ** *p* < 0.05.

Since endogenous ET_A_R and ET_B_R were previously detected in HeLa cell lines [23], we tested whether Sal A inhibited endogenous ET_A_R and ET_B_R. Stimulation of HeLa cells with ET-1 led to a concentration-dependent, time-resolved impedance response as measured by the xCelligence system [24]. In our study, after 24 h incubation with complete medium, the medium was changed to hanks balanced salt solution (HBSS) with Ca^2+^ and Mg^2+^. Then, various concentrations of Sal A, 10 µM Bosentan (positive control), and 0.1% DMSO (negative control) were performed in parallel. A rapid impedance response was observed as cell index (CI) decreased and recovered within 15 min after initial treatment. Subsequently, 10 nM ET-1 was added. Cell index was sharply elevated by ET-1, and the response was completely blocked by Bosentan. Sal A also declined ET-1-induced changes of CI and showed a dose-dependent trend (Figure 4A). The concentration–response curve was calculated based on (Max_CI_ − Min_CI_) during 29.7 to 30.4 h, and the IC_50_ value of Sal A was 3.14 µM (Figure 4B). Furthermore, the effects of Bosentan and Sal A in HeLa cells were investigated by Ca^2+^ Influx assay. ET-1 dose-dependently induced calcium mobilization from 500 nM to 6.4 pM with an EC_50_ value of ET-1 of 39 nM (Figure 4C). This response was fully blocked by Bosentan and the IC_50_ was 4.3 µM (Figure 4D). In contrast, Sal A exhibited partial antagonism of endogenous ET_A_R, with IC_50_ of 5.19 µM (Figure 4E). These responses were further confirmed by analysis of respond-span, in which Bosentan completely and Sal A partially suppressed (*p* < 0.01) ET-1 activation (Figure 4F). These results suggested that Sal A could block both recombinant and endogenous ET_A_R.

### 2.5. Sal A Suppressed Proliferations in Multiple Cancer Cell Lines

As indicated in Table 1, anti-proliferative effects were tested in five cell lines—including human ovarian carcinoma cells (SKOV3), human cervical cancer cells (HeLa), human non-small cell lung cancers with T790 mutation (NCI-H1975), human prostate carcinoma cells (DU145), human lung adenocarcinoma cells (A549)—with Sal A treatment at various concentrations of 100, 25, 6.25, and 1.56 µM. After 72 h treatment, cell viability was determined by CellTiter-Glo kit. With the increase of Sal A concentration, cell growth was suppressed in all cell lines. However, different IC_50_ values were observed in each cell line.

### 2.6. Sal A Extended Cell Lins Proliferation Cycle by Real-Time Cell Analysis (RTCA)

**Figure 5:**
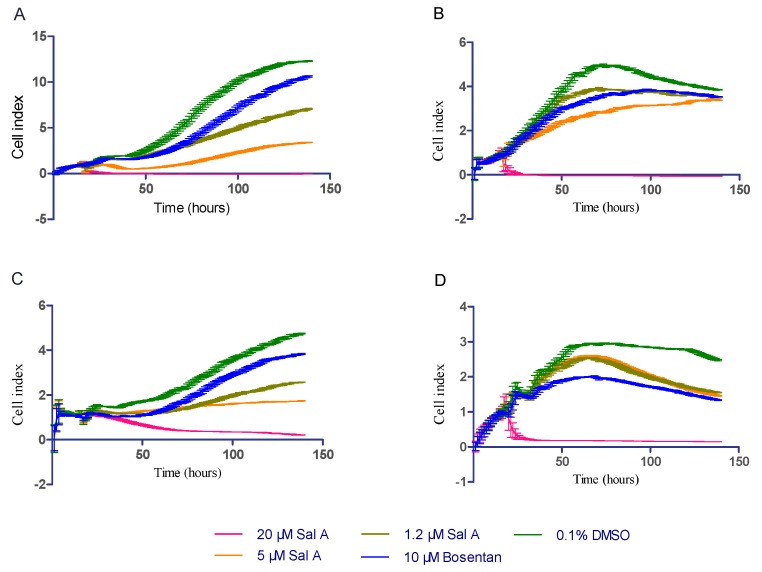
Real-time monitoring of cell proliferation using the xCELLigence system. (**A**) DU145; (**B**) HeLa; (**C**) H1975; and (**D**) A549 cell lines were seeded with 6000 cells per well onto E-plate. After 16 h, cells were cultured in reduced serum medium (2% FBS) in the presence of 20, 5, 1.2 µM Sal A, 10 µM Bosentan, and 0.1% DMSO as negative control. CI was monitored continuously for 130 h. The xCELLigence system was set to record the cell index every 70 min. Data were expressed as mean and SD in two independent experiments.

To characterize the mechanism of Sal A, we assessed the effect of Sal A and Bosentan on DU145, A549, H1975, HeLa cell lines by using the xCELLigence system. After cell seeding onto 96 well E-plate for 16 h, cells were cultured in reduced serum medium (2% FBS) in the presence of 20, 5, 1.2 µM Sal A, and 10 µM Bosentan, and 0.1% DMSO as negative control. Relative impedance signal level (represented as “cell index” in manufacturer’s software) was monitored continuously for 130 h. The xCELLigence system were set to record the cell index every 70 min. All cell lines reach a stationary phase of growth in 80 to 130 h (Figure 5). With the treatment of 10 µM Bosentan, the time of reaching stationary phase was extended in four cell lines. Similarly, 5 and 1.2 µM Sal A extended the proliferation time. Meanwhile, cell indexes of four cell lines induced by 20 µM Sal A were detected to decrease, in which the concentration was lower than that we tested in the CellTiter-Glo assay.

To compare the inhibitory effect in quantification, we calculated the doubling time (DT) index by using Formula 1. Based on the exponent proliferation curve, we estimated the doubling time by using cell index from 16 h to the time point of maximum cell index.

Time indicates the time from starting to the time of maximum cell index. The parameter estimation was by Prism 6 (GraphPad Software, La Jolla, CA, USA), initial values were 1. The results were output, including the value of doubling time (DT) and 95% confidence interval of DT.

(1) $$\text{Cell Index}=A\times 2^{\text{Time}/\text{DT}}$$

The results are shown in Table 2. Ten µM Bosentan extended the doubling time of four cell lines significantly. The extension of doubling time was observed with the treatment of 5 and 1.2 µM Sal A. The doubling times of HeLa, DU145, and H1975 were significantly more than negative control, but A549 cell lines showed no influence of Sal A on doubling time. Interestingly, Sal A induced a cell index decrease after 60 h in A549 cell lines. The influence of Sal A in A549 cell lines should be further investigated.

### 2.7. Sal A Inhibited the Proliferation Induced by Exogenous ET-1 in DU145 Cells

**Figure 6:**
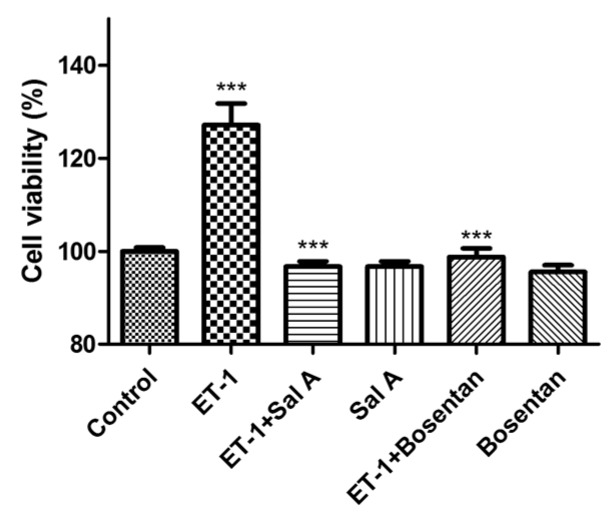
Growth response of prostate cancer cell lines to ET-1 (10 nM) and Sal A (10 µM, ET_A_ receptor antagonist) and Bosentan (10 µM, ET_A_, and ET_B_ receptor antagonist). After 48 h incubation with compounds, cell viability (%) was compared with controls (set at 100%). Bosentan and Sal A significantly reduced growth in the presence of exogenous ET-1 (vs. ET-1 group). Without the presence of exogenous ET-1, both Bosentan and Sal A showed no inhibitory effect in DU145 (vs. Control). Data were expressed as mean and SD of three independent experiments. Statistical analysis of original RLU measurement was conducted using one-way ANOVA with Tukey test. *** *p* < 0.001.

The ability of Sal A—an ET_A_ receptor antagonist—to inhibit ET-1 growth stimulation was tested in DU145 cells. Growth effects were determined independently in both cell lines in the presence of ET-1 alone, ET-1, ET_A_ and ET_B_ receptor antagonists (Sal A, Bosentan), and all antagonists alone (48 h). As shown in Figure 6, exogenous 10 nM ET-1 induced an increase in proliferation of approximately 30%. Meanwhile, ET-1-stimulated growth was significantly blocked by Bosentan (ET_A_R and ET_B_R antagonist) and Sal A. Incubation with the compounds alone did not significantly affect cell growth.

### 2.8. Effect of Sal A on Cell Cytotoxicity in HUVEC

**Figure 7:**
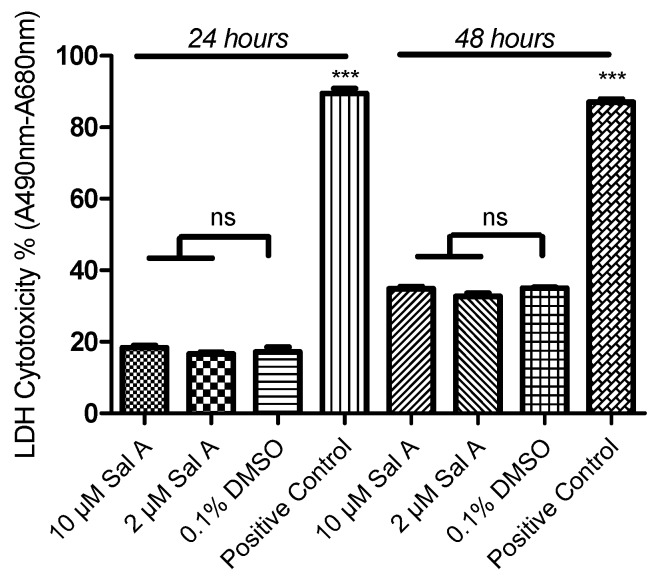
Determination of lactate dehydrogenase (LDH) cytotoxicity of Sal A in HUVEC cells. HUVEC cells were plated in a 96-well plate in maintaining medium. Different concentrations of Sal A were added to the culture media and incubated for 24 and 48 h at 37 °C, 5% CO_2_. Meanwhile, 1X Lysis Buffer was used as positive control. LDH cytotoxicity was measured using the Pierce LDH Cytotoxicity Assay Kit (Thermo Scientific). Data were expressed as mean ± SD, *n* = 3. Statistical analysis of original optical density value measurement was conducted using a one-way ANOVA and a with Tukey test. *** *p* < 0.001. ns: no significant difference.

We investigated the effect of Sal A on cell cytotoxicity in HUVEC cells. As shown in Figure 7, released lactate dehydrogenase (LDH) cytotoxicity was evaluated in this experiment. HUVEC cells were treated with 10 and 2 µM of Sal A for 24 and 48 h. LDH cytotoxicity was measured using the Pierce LDH Cytotoxicity Assay Kit (Thermo Scientific, Hudson, NH, USA). The results indicated that there are no significant differences in extracellular LDH between test and control groups in both 24 and 48 h.

### 2.9. Effect of Sal A on Cardio-Toxicity in hiPS-CMs

**Figure 8:**
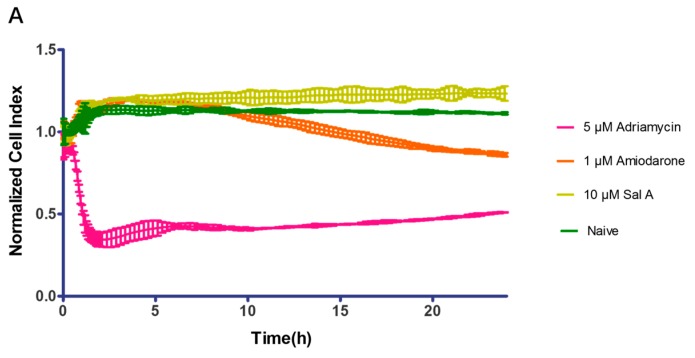

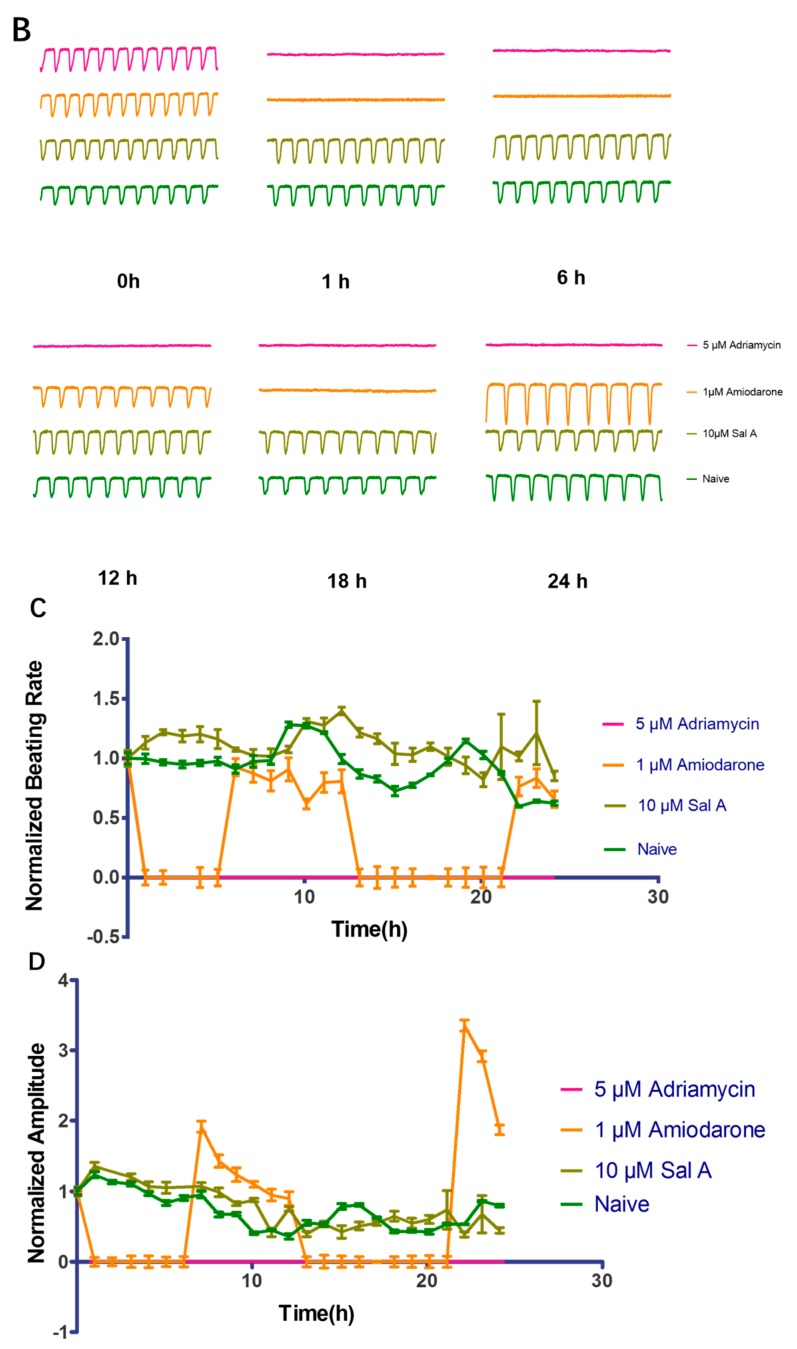
Typical contraction profiles of human-induced pluripotent stem cell-derived cardiomyocytes (hiPS-CMs) to compounds treatment. (**A**) Cell index fluctuation after Adriamycin (5 µM, cytotoxicity positive control), Amiodarone (1 µM, cardio-toxicity control), and Sal A (10 µM); (**B**) Temporal profiling of hiPS-CMs beating pattern. Beating pattern data were expressed in 0, 1, 6, 12, 18, 24 h; (**C**) Temporal profiling of hiPS-CMs beating rate. Data were normalized by the point before compounds were added; (**D**) Temporal profiling of hiPS-CMs amplitude. Data were normalized to the signal acquired prior to compound treatment. Data were analyzed using xCELLigence Cardio Software (ACEA, Hangzhou, China) and expressed as mean ± SD of three independent experiments.

The xCelligence RTCA cardio system has been a useful tool used to monitor the cardio-toxicity effect on hiPS-CMs in the short and long term [25,26]. In this study, the effect of Sal A on available commercial hiPS-CMs was evaluated. Cells were treated with compounds post cell seeding for 24 h. As shown in Figure 8A, cell index remained stable after treatment with Sal A. Meanwhile, Adriamycin (5 µM) as the positive control decreased the cell index, indicating that Sal A induced no cell death.

At the same time, we monitored the effect of Sal A on hiPS-CMs contractility activities in real-time. Before compound treatment, hiPS-CMs contractility was detected every minute for at least 10 min to confirm the stability of the hiPS-CMs. In Figure 8B–D, 10 µM Sal A had no inhibitory effect on hiPS-CMs beating rates and amplitude in the long term. Simultaneously, amiodarone in 1 µM was treated as positive control for inducing cardio-toxicity in hiPS-CMs. As shown in Figure 8B, the amiodarone (positive control) decreased beating rate within 10 h, and the recovery of hiPS-CMs contractility activities were observed after 10 h. The decreasing contractility activities indicated cardio-toxicity [27]. Compared with amiodarone, the contractility activities were not influenced by 10 µM Sal A. Above all, these results suggested that 10 µM Sal A was free of cardio-toxicity and cell toxicity on hiPS-CMs with 24 h.

## 3. Discussion

Salvianolic acid derivatives are the major water-soluble components of Dashen, which is the dried roots and rhizomes of *Salvia miltiorrhiza* Bunge, a type of well-known traditional Chinese herbal medicine. Among various Salvianolic acids, Salvianolic acid A has drawn considerable research attention for its diverse potent bioactivities, including the suppressive effect of transgelin 2 and the inhibitory effect of matrix metalloproteinase-9 [20,28]. In our study, we demonstrate for the first time that Sal A is a selective ET_A_R antagonist in both exogenous and endogenous cell lines and reveal the inhibitory effect of Sal A on the proliferation of multiple tumor cell lines. Moreover, we traced the non-cytotoxicity and non-cardio-toxicity of Sal A in HUVECs and hiPS-CMs.

In the ET_A_ receptor-overexpressed HEK293 cell line, we observed the dose-dependent inhibitory effect of Sal A. The results of the selective assay suggested that Sal A possessed a selective inhibitory effect on ET_A_ receptors. The similar inhibitory effect was observed after treatment of serial concentration of Sal A in HeLa cell line. By using RTCA, we determined that Sal A inhibited the Cell Index increase induced by ET-1 partially, and the CI increase was completely suppressed by Bosentan. In the Ca^2+^ influx assay (Figure 4C–F), the dose–response span of Sal A was less than Bosentan. The results demonstrated that Sal A inhibits ET_A_R activation and suggested Sal A as a functional antagonist of ET_A_R. The potential binding mechanism of Sal A to the endothelin receptor will be evaluated in further research.

The ET-1–ET_A_R axis has been widely involved in the cancer process. ET_A_R antagonists, such as Zibotentan and Atrasentan, were investigated in a series of cancer therapy clinical trials [29,30]. Thus, several cancer cell lines, including HeLa, SKOV3, DU145, H1975, and A549—all of which expressed ET_A_R [31,32,33,34]—were applied to investigations of the anti-proliferative effects of Sal A. By CellTiter-Glo kit and RTCA assays, Sal A showed anti-proliferative effects in HeLa, DU145, H1975, and A549 cell lines with IC_50_ values under 20 µM. During the continuous monitor proliferation assay, both Sal A and Bosentan delayed the increase of cell index. However, IC_50_ values between ET_A_R antagonist and anti-tumor assays showed a remarkable difference (approximately 3 µM for antagonism assay and up to 15 µM for the anti-proliferation assay). A possible explanation for this variation may lie in the differences in detection approaches and cell lines. As shown in Figure 5D, both Sal A and Bosentan induced a cell index decrease after 60 h in A549 cell lines. In the study of Bi, L. [35], Sal A induced A549 cell lines apoptosis by up-regulating the phosphatase and tensin homolog (PTEN) protein level and down-regulating Akt phosphorylation. The doubling time index was used in our study for the comparison of the effect of Sal A in four cell lines. In the study of Masarik, M. [36], the slope of the cell index increase curve was used to describe the proliferation phenomena in the RTCA assay. Based on a similar principle, we employed the simple doubling time formula to estimate the doubling time. In brief, we estimated the parameter of Formula 1 by using the cell index from the time of adding compounds to the time point of maximum cell index. Furthermore, we demonstrated that Sal A suppresses the proliferation induced by exogenous ET-1 in DU145 cells (shown in Figure 6). Ping Sun [32] reported that exogenous ET-1 reduces the apoptosis induced by paclitaxel in DU145 cells. Generally, the majority of cancer cell types—for example, prostate, ovarian, and lung cancers—show a reduction of ET-1-stimulated growth in response to ET_A_R antagonism [12,37,38]. These findings suggested that Sal A may play a role in cancer therapy through inhibition of ET_A_R activation.

Advances in cancer therapy have resulted in significant improvement in long-term survival for many types of cancer, but have also resulted in untoward side effects associated with treatment. One such complication that has become increasingly recognized is the development of cardiomyopathy and heart failure [39,40]. Although Sal A is reported to attenuate H9C2 cell apoptosis [41], the potential toxic risk of Sal A on HUVEC and hiPS-CMs at bioactive doses still remains to be explored. LDH release assay showed that Sal A did not show cytotoxicity to HUVECs at a concentration of 10 µM during a 48 h period. Compared to cytotoxicity assays on H9C2 [42], the RTCA cardio system provides a sensitive tool to predict potential cardiac side effects. Additionally, the sensitive label-free assay made it possible to detect the regular beating pattern of cardiomyocytes under normal physiological conditions [42,43,44]. As mentioned [26], the loss of cell index reflected the loss of mitochondria and cell viability induced by Adriamycin. Amiodarone lengthens the cardiac action potential to induce acute cardiac myocyte dysfunction [45]. Cytotoxicity of hiPS-CMs was clearly shown to be induced by 5 µM Adriamycin with a cell index decrease, but not by 10 µM Sal A. Treatment with 10 µM Sal A did not induce modification of amplitude nor beat rate compared with vehicle control. These observations suggest that Sal A is above the cardio-toxic threshold and provide a cardio-friendly anti-cancer leading compound.

## 4. Materials and Methods

### 4.1. Reagents and Materials

Dulbecco’s modified eagles medium (DMEM) and fetal bovine serum (FBS) for cell culture were purchased from Gibco BRL (Grand Island, NY, USA). Salvianolic acid A, Salvianolic acid B, Salvianolic acid C, Salvianolic acid D, and Salvianolic acid were purchased from National Institutes for Food and Drug Control (Beijing, China) with purities greater than 98%. Hygromycin B, ET-1, Angiotensin II, Adenosine, TRAP-6, probenecid, and acid red 1 (purity ≥ 98%) were purchased from Sigma-Aldrich Chemicals (St. Louis, MO, USA). Bosentan hydrate was purchased from Yuanye (Shanghai, China). Fluo-4 AM was purchased from Molecular Probes (Grand Island, NY, USA). Matrigel was purchased from Becton Dickinson (New York, NY, USA). All the chemicals were dissolved in DMSO if not otherwise stated.

### 4.2. Compund Preparation

Salvianolates were dissolved in DMSO at 36 mM. For primary screening, compounds were diluted to 12 mM in DMSO. ET-1 and BQ-123 were stored at a concentration of 50 µM and 1 mM, respectively. Initial serial dilutions were made in DMSO with compound concentrations at 400× final for concentration-response determinations. For the compound plates, 2 µL of the 400× DMSO solution was added to 160 µL HBSS (5× solution, DMSO concentration 1.25%). The final DMSO concentration in each well was controlled as 0.25% for all the tested compounds.

### 4.3. Cell Culture

HEK293/ET_A_R cells, provided by the Beijing Institute of Genomics in the Chinese Academy of Science (Beijing, China), were well-characterized cell lines expressing the ET_A_ receptor. All HEK293 cell lines used in this study were routinely maintained in DMEM containing 10% FBS, 100 U/mL penicillin, and 100 µg/mL streptomycin in a humidified atmosphere of 5% CO_2_ at 37 °C. HEK293/ET_A_R cells were incubated with complete culture medium along with 50 µg/mL hygromycin B. HeLa, A549, SKOV3, DU145, and H1975 cell lines were maintained in DMEM containing 10% FBS, 100 U/mL penicillin, and 100 µg/mL streptomycin. The hiPS-CMs cells were obtained from CELLAPYBIO (Cat# CA2001106, Beijing, China), which are a well-validated cell line [46], and CMs were thawed from cryopreserved vials into CMs plating medium following recommended procedures. The cells were incubated in DMEM with 10% FBS at 37 °C, 5% CO_2_. Cell culture media were refreshed every two days. HUVEC cells, a gift from Zengchangqing Lab, were maintained in EBM-2 medium with SingleQuot growth kit (Lonza, Allendale, NJ, USA).

### 4.4. Antagonist Screening Assay

#### 4.4.1. Ca^2+^ Influx Assay

HEK293/ET_A_R cells were seeded at a density of 3 × 10^4^ per well into 96-well clear-bottom black plates coated with matrigel and incubated in 5% CO_2_ at 37 °C overnight. On the day of assay, the growth medium was replaced by 80 µL loading buffer containing a final concentration of 4 µM Ca^2+^-sensitive dye Fluo-4 AM and 2 mM acid red 1 in HBSS. The plate was then incubated at 37 °C in the dark for 30 min before calcium signal read out. For antagonist study, 80 µL loading buffer was added into each well and 20 µL HBSS containing tested compound was added 10 min prior to calcium-flux measurement. Cells were transferred to a Flexstation II (Molecular Devices, Sunnyvale, CA, USA) for experimentation. Basal fluorescence was recorded for 16 s before agonist application. The integrated Flexstation II fluidics system added 25 µL compound (5× solution) from the agonist compound plate to the assay plate containing 100 µL loading buffer solution. Relative fluorescence units (RFU) were read by FlexStation II at 37 °C with an excitation wavelength of 485 nm and an emission wavelength of 525 nm. The fluorescence intensity was read every 1.52 s for 80 s. RFU indicated the peak of calcium response. The inhibition (%) were calculated as follows,

(2) $$\text{Inhibition}\;\left(\%\right)=\left(1-\frac{{RFU}_{\text{compound}}-{RFU}_{\text{negetive}}}{{RFU}_{\text{positive}}-{RFU}_{\text{negetive}}}\right)\times 100\%$$

#### 4.4.2. Real-Time Cell Analysis Assay

HeLa cell lines were seeded at a density of 1 × 10^4^ per well into a 96-well E-plate and the impedance was monitored by xCELLgence system in 5% CO_2_ at 37 °C overnight. On the day of the assay, the growth medium was replaced by 80 µL HBSS. After 30 min, the 20 µL compound (5× solution) were added into the E-plate. Then, the 25 µL ET-1 (5× solution) were added into the E-plate when the cell indexes were steady. The cell index was read every 20 s for 1 h.

### 4.5. Compound Cytotoxicity Assay

#### 4.5.1. Cell Viability Assay

HEK293/ET_A_R cells were seeded at 3 × 10^4^ per well into 96-well clear-bottom black plates and incubated in 5% CO_2_ at 37 °C overnight. Different concentrations of the compound were added into the 96-well plates and incubated for 2 h. Luminescence was read by Envision 2100 multilabel reader to detect cells’ viability following incubation with CellTiter-Glo reagent for 10 min.

#### 4.5.2. Lactate Dehydrogenase Leakage Assay

HUVEC cells (1.5 × 10^4^ cells/well) were seeded in a 96-well plate. The cultured cells were treated with various concentrations of compounds or vehicle and were incubated for another 24 and 48 h. The culture medium was aspirated and centrifuged at 1000× *g* for 10 min to obtain a cell-free supernatant. LDH activity was examined using a commercially available kit (Thermo Fisher Scientific, Pittsburgh, PA, USA) following the manufacturer’s instructions. These measurements were performed with VersaMax (Molecular Devices, Sunnyvale, CA, USA). The results are given as fractions of LDH release compared to the positive controls, which consisted of Lysis Buffer. The results presented represent mean values from triplicate measurements.

#### 4.5.3. Cardio-Toxicity Assay

The hiPS-CM were seeded at 1.7 × 10^4^ in 96-well E-plate. Procedures have been described in [25]. In brief, compound treatment was initiated 48–72 h after cell seeding, and the E-plate was monitoring every 15 min on the RTCA Cardio Instrument incubator. The concentration of DMSO were control in 0.1%.

### 4.6. Cell Proliferation Assay

#### 4.6.1. Cell Viability Assay

Cell lines were seeded at 5 × 10^3^ per well into 96-well plates and incubated in 5% CO_2_ at 37 °C overnight. On the day of assay, the cell culture medium was replaced with 108 µL complete culture medium within 10% FBS. The 12 µL of different concentrations of Salvianolic A (10× solution) were added into the 96-well plates and incubated for 72 h. The concentration of DMSO was controlled at 0.1%. Luminescence was read by Envision 2100 multilabel reader to detect cells’ viability following incubation with CellTiter-Glo reagent for 10 min.

#### 4.6.2. Real-Time Cell Analysis Assay

Cell lines were seeded at 5 × 10^3^ per well into 96-well E-plates and incubated in 5% CO_2_ at 37 °C overnight. Then, the cell culture medium was replaced with 108 µL reduced serum medium within 2% FBS. The 12 µL of different concentrations of compounds (10× solution) were added into the 96-well E-plates and incubated for 138 h. The cell index was read every 70 min.

### 4.7. Data Analysis and Statistics

Data were analyzed using xCELLigence Cardio Software (Roche, Basel, Switzerland) and further analyzed with GraphPad Prism 6. Data were presented as mean ± SD. Statistical significance of differences was estimated by one-way ANOVA. *p* < 0.05 (marked with an asterisk) was considered significant.

## 5. Conclusions

In conclusion, we found that Sal A is a novel ET_A_ receptor antagonist, and further observed anti-tumor effect with or without exogenous endothelin-1. Our data strongly suggest that Sal A is a potential candidate for the development of a novel anti-cancer drug.

## Figures and Tables

**Table 1:** Anti-proliferative effects and IC_50_ values of salvianolic acid A (Sal A).

Cell Lines	Inhibition (%)				IC_50_ Values (µM)
	100 µM	25 µM	6.25 µM	1.56 µM	
SKOV3	99.13(0.19)	30.87(3.18)	1.215(1.48)	6.001(0.21)	30.84
HeLa	96.30(0.49)	75.65(3.81)	8.751(1.25)	3.419(0.15)	15.85
H1975	96.38(0.19)	73.65(1.84)	35.09(5.75)	11.64(1.48)	10.19
DU145	96.48(0.04)	77.25(2.18)	32.94(2.26)	18.15(4.08)	9.512
A549	99.13(0.11)	58.43(2.39)	43.40(1.55)	38.35(2.56)	6.461

The anti-proliferative effect of Sal A on SKOV3, HeLa, H1975, DU145, and A549 cell lines. Cells were treated with Sal A at doses of 100, 25, 6.25, and 1.56 µM. After 72 h, cell viability was determined by CellTiter-Glo kit (Promega Co., Madison, WI, USA), and the inhibitions (%) were calculated (vs. Naïve group). The concentration of DMSO was controlled at 0.1%. Data are expressed as mean (SD) from three independent experiments.

**Table 2:** The effect of Sal A on doubling time in different cell lines (h).

Cell Lines	Sal A	Sal A	Bosentan	0.1% DMSO
	5 µM	1.2 µM	10 µM	
HeLa	33.5 ± 1.04 **	23.7 ± 0.48 **	26.3 ± 0.62 **	21.1 ± 0.34
DU145	41.0 ± 1.35 **	35.8 ± 0.37 **	29.6 ± 0.26 **	25.6 ± 0.19
H1975	94.1 ± 2.43 **	49.6 ± 1.61 **	29.4 ± 0.38 *	27.1 ± 0.63
A549	28.7 ± 1.10	28.9 ± 1.08	40.9 ± 2.33 **	29.7 ± 0.99

The effect of Sal A on doubling time in HeLa, H1975, DU145, A549 cells line. Cells were treated with Sal A at a series of concentrations. The doubling time was calculated during 16 to 80 h. The concentration of DMSO was controlled at 0.1%. Data are expressed as mean ± 95% confidence interval. Statistical analysis was conducted using one-way ANOVA with Tukey test. * *p* < 0.1; ** *p* < 0.05.

## Supplementary Materials

Supplementary materials can be found at http://www.mdpi.com/1422-0067/17/8/1244/s1.

## References

[1] Grant K., Loizidou M., Taylor I. (2003). Endothelin-1: A multifunctional molecule in cancer. Br. J. Cancer.

[2] Rosano L., Spinella F., Bagnato A. (2013). Endothelin 1 in cancer: Biological implications and therapeutic opportunities. Nat. Rev. Cancer.

[3] Shichiri M., Hirata Y., Nakajima T., Ando K., Imai T., Yanagisawa M., Masaki T., Marumo F. (1991). Endothelin-1 is an autocrine/paracrine growth factor for human cancer cell lines. J. Clin. Investig..

[4] Nelson J., Bagnato A., Battistini B., Nisen P. (2003). The endothelin axis: emerging role in cancer. Nat. Rev. Cancer.

[5] Jin J.K., Dayyani F., Gallick G.E. (2011). Steps in prostate cancer progression that lead to bone metastasis. Int. J. Cancer.

[6] Nie S., Zhou J., Bai F., Jiang B., Chen J., Zhou J. (2014). Role of endothelin A receptor in colon cancer metastasis: In vitro and in vivo evidence. Mol. Carcinog..

[7] Bagnato A., Cirilli A., Salani D., Simeone P., Muller A., Nicotra M.R., Natali P.G., Venuti A. (2002). Growth inhibition of cervix carcinoma cells in vivo by endothelin A receptor blockade. Cancer Res..

[8] Banerjee S., Hussain M., Wang Z., Saliganan A., Che M., Bonfil D., Cher M., Sarkar F.H. (2007). In vitro and in vivo molecular evidence for better therapeutic efficacy of ABT-627 and taxotere combination in prostate cancer. Cancer Res..

[9] Haque S.U., Dashwood M.R., Heetun M., Shiwen X., Farooqui N., Ramesh B., Welch H., Savage F.J., Ogunbiyi O., Abraham D.J. (2013). Efficacy of the specific endothelin a receptor antagonist zibotentan (ZD4054) in colorectal cancer: A preclinical study. Mol. Cancer Ther..

[10] Daub H., Weiss F.U., Wallasch C., Ullrich A. (1996). Role of transactivation of the EGF receptor in signalling by G-protein-coupled receptors. Nature.

[11] Grant K., Knowles J., Dawas K., Burnstock G., Taylor I., Loizidou M. (2007). Mechanisms of endothelin 1-stimulated proliferation in colorectal cancer cell lines. Br. J. Surg..

[12] Akhavan A., McHugh K.H., Guruli G., Bies R.R., Zamboni W.C., Strychor S.A., Nelson J.B., Pflug B.R. (2006). Endothelin receptor A blockade enhances taxane effects in prostate cancer. Neoplasia.

[13] Rosano L., Di Castro V., Spinella F., Tortora G., Nicotra M.R., Natali P.G., Bagnato A. (2007). Combined targeting of endothelin A receptor and epidermal growth factor receptor in ovarian cancer shows enhanced antitumor activity. Cancer Res..

[14] Liu T., Liu X., Li W. (2016). Tetrandrine, a Chinese plant-derived alkaloid, is a potential candidate for cancer chemotherapy. Oncotarget.

[15] Apaya M.K., Chang M.T., Shyur L.F. (2016). Phytomedicine polypharmacology: Cancer therapy through modulating the tumor microenvironment and oxylipin dynamics. Pharmacol. Ther..

[16] Moselhy J., Srinivasan S., Ankem M.K., Damodaran C. (2015). Natural products that target cancer stem cells. Anticancer Res..

[17] Fan H.Y., Fu F.H., Yang M.Y., Xu H., Zhang A.H., Liu K. (2010). Antiplatelet and antithrombotic activities of salvianolic acid A. Thromb. Res..

[18] Liu G.T., Zhang T.M., Wang B.E., Wang Y.W. (1992). Protective action of seven natural phenolic compounds against peroxidative damage to biomembranes. Biochem. Pharmacol..

[19] Oh K.S., Oh B.K., Mun J., Seo H.W., Lee B.H. (2011). Salvianolic acid A suppress lipopolysaccharide-induced NF-κB signaling pathway by targeting IKKβ. Int. Immunopharmacol..

[20] Zheng X., Chen S., Yang Q., Cai J., Zhang W., You H., Xing J., Dong Y. (2015). Salvianolic acid A reverses the paclitaxel resistance and inhibits the migration and invasion abilities of human breast cancer cells by inactivating transgelin 2. Cancer Biol. Ther..

[21] Cai J., Chen S., Zhang W., Zheng X., Hu S., Pang C., Lu J., Xing J., Dong Y. (2014). Salvianolic acid A reverses paclitaxel resistance in human breast cancer MCF-7 cells via targeting the expression of transgelin 2 and attenuating PI3 K/Akt pathway. Phytomed. Int. J. Phytother. Phytopharm..

[22] Ihara M., Ishikawa K., Fukuroda T., Saeki T., Funabashi K., Fukami T., Suda H., Yano M. (1992). In vitro biological profile of a highly potent novel endothelin (ET) antagonist BQ-123 selective for the ETA receptor. J. Cardiovasc. Pharmacol..

[23] Drimal J., Drimal J., Drimal D. (2000). Enhanced endothelin ET(B) receptor down-regulation in human tumor cells. Eur. J. Pharmacol..

[24] Ke N., Nguyen K., Irelan J., Abassi Y.A. (2015). Multidimensional GPCR profiling and screening using impedance-based label-free and real-time assay. Methods Mol. Biol..

[25] Yu Y., Sun S., Wang S., Zhang Q., Li M., Lan F., Li S., Liu C. (2016). Liensinine- and neferine-induced cardiotoxicity in primary neonatal rat cardiomyocytes and human-induced pluripotent stem cell-derived cardiomyocytes. Int. J. Mol. Sci..

[26] Clements M., Millar V., Williams A.S., Kalinka S. (2015). Bridging functional and structural cardiotoxicity assays using human embryonic stem cell-derived cardiomyocytes for a more comprehensive risk assessment. Toxicol. Sci..

[27] Nguemo F., Saric T., Pfannkuche K., Watzele M., Reppel M., Hescheler J. (2012). In vitro model for assessing arrhythmogenic properties of drugs based on high-resolution impedance measurements. Cell. Physiol. Biochem. Int. J. Exp. Cell. Physiol. Biochem. Pharmacol..

[28] Zhang T., Xu J., Li D., Chen J., Shen X., Xu F., Teng F., Deng Y., Ma H., Zhang L. (2014). Salvianolic acid A, a matrix metalloproteinase-9 inhibitor of Salvia miltiorrhiza, attenuates aortic aneurysm formation in apolipoprotein E-deficient mice. Phytomed. Int. J. Phytother. Phytopharm..

[29] Chouaid C., Nathan F., Pemberton K., Morris T. (2011). A phase II, randomized, multicenter study to assess the efficacy, safety, and tolerability of zibotentan (ZD4054) in combination with pemetrexed in patients with advanced non-small cell lung cancer. Cancer Chemother. Pharmacol..

[30] Nelson J.B., Love W., Chin J.L., Saad F., Schulman C.C., Sleep D.J., Qian J., Steinberg J., Carducci M. (2008). Atrasentan Phase 3 Study Guides Phase 3, randomized, controlled trial of atrasentan in patients with nonmetastatic, hormone-refractory prostate cancer. Cancer.

[31] Chauhan V., Breznan D., Thomson E., Karthikeyan S., Vincent R. (2005). Effects of ambient air particles on the endothelin system in human pulmonary epithelial cells (A549). Cell Biol. Toxicol..

[32] Sun P., Xiong H., Kim T.H., Ren B., Zhang Z. (2006). Positive inter-regulation between β-catenin/T cell factor-4 signaling and endothelin-1 signaling potentiates proliferation and survival of prostate cancer cells. Mol. Pharmacol..

[33] Von Brandenstein M.G., Ngum Abety A., Depping R., Roth T., Koehler M., Dienes H.P., Fries J.W. (2008). A p38-p65 transcription complex induced by endothelin-1 mediates signal transduction in cancer cells. Biochim. Biophys. Acta.

[34] Tocci P., Caprara V., Cianfrocca R., Sestito R., Di Castro V., Bagnato A., Rosano L. (2016). Endothelin-1/endothelin A receptor axis activates RhoA GTPase in epithelial ovarian cancer. Life Sci..

[35] Bi L., Chen J., Yuan X., Jiang Z., Chen W. (2013). Salvianolic acid A positively regulates PTEN protein level and inhibits growth of A549 lung cancer cells. Biomed. Rep..

[36] Masarik M., Gumulec J., Hlavna M., Sztalmachova M., Babula P., Raudenska M., Pavkova-Goldbergova M., Cernei N., Sochor J., Zitka O. (2012). Monitoring of the prostate tumour cells redox state and real-time proliferation by novel biophysical techniques and fluorescent staining. Integr. Biol. Quant. Biosci. Nano Macro.

[37] Kuzumaki N., Suzuki A., Narita M., Hosoya T., Nagasawa A., Imai S., Yamamizu K., Morita H., Suzuki T., Okada Y. (2012). Multiple analyses of G-protein coupled receptor (GPCR) expression in the development of gefitinib-resistance in transforming non-small-cell lung cancer. PLoS ONE.

[38] Rosano L., Cianfrocca R., Spinella F., di Castro V., Natali P.G., Bagnato A. (2010). Combination therapy of zibotentan with cisplatinum and paclitaxel is an effective regimen for epithelial ovarian cancer. Can. J. Physiol. Pharmacol..

[39] Bloom M.W., Hamo C.E., Cardinale D., Ky B., Nohria A., Baer L., Skopicki H., Lenihan D.J., Gheorghiade M., Lyon A.R. (2016). Cancer therapy-related cardiac dysfunction and heart failure: Part 1: Definitions, pathophysiology, risk factors, and imaging. Circ. Heart Fail..

[40] Hamo C.E., Bloom M.W., Cardinale D., Ky B., Nohria A., Baer L., Skopicki H., Lenihan D.J., Gheorghiade M., Lyon A.R. (2016). Cancer therapy-related cardiac dysfunction and heart failure: Part 2: Prevention, treatment, guidelines, and future directions. Circul. Heart Fail..

[41] Fan H., Yang L., Fu F., Xu H., Meng Q., Zhu H., Teng L., Yang M., Zhang L., Zhang Z. (2012). Cardioprotective effects of salvianolic Acid a on myocardial ischemia-reperfusion injury in vivo and in vitro. Evid. Based Complement. Altern. Med. eCAM.

[42] Choi E.H., Chang H.J., Cho J.Y., Chun H.S. (2007). Cytoprotective effect of anthocyanins against doxorubicin-induced toxicity in H9c2 cardiomyocytes in relation to their antioxidant activities. Food Chem. Toxicol. Int. J. Publ. Br. Ind. Biol. Res. Assoc..

[43] Eldridge S., Guo L., Mussio J., Furniss M., Hamre J., Davis M. (2014). Examining the protective role of ErbB2 modulation in human-induced pluripotent stem cell-derived cardiomyocytes. Toxicol. Sci..

[44] Guo L., Eldridge S., Furniss M., Mussio J., Davis M. (2015). Use of human induced pluripotent stem cell-derived cardiomyocytes (hiPSC-CMs) to monitor compound effects on cardiac myocyte signaling pathways. Curr. Protoc. Chem. Biol..

[45] Guo L., Coyle L., Abrams R.M., Kemper R., Chiao E.T., Kolaja K.L. (2013). Refining the human iPSC-cardiomyocyte arrhythmic risk assessment model. Toxicol. Sci..

[46] Liang P., Lan F., Lee A.S., Gong T., Sanchez-Freire V., Wang Y., Diecke S., Sallam K., Knowles J.W., Wang P.J. (2013). Drug screening using a library of human induced pluripotent stem cell-derived cardiomyocytes reveals disease-specific patterns of cardiotoxicity. Circulation.

