# Supplementary material for: Salvianolic Acid A, as a Novel ETA Receptor Antagonist, Shows Inhibitory Effects on Tumor in Vitro
Source: Int J Mol Sci. 2016 Aug 2;17(8):1244. doi: 10.3390/ijms17081244 (PMC5000642; doi:10.3390/ijms17081244)
Supplement: Supplementary file 1 [file ijms-17-01244-s001.pdf]

Supplementary Materials: Salvianolic Acid A, as a Novel ETA Receptor Antagonist, Shows Inhibitory Effects on Tumor in Vitro

Qiao Zhang, Shifeng Wang, YangyangYu, Shengnan Sun, Yuxin Zhang, Yanling Zhang, Wei Yang, Shiyou Li and Yanjiang Qiao

Table S1. The cell lines and compounds in the selectivity assay.

Cell Lines	Agonists	EC_{50}	Concentration in Selectivity Assay	Antagonists	Concentration in Selectivity Assay
HEK293/ET_AR	ET-1	4.78 nM	20 nM	Bosentan	10 μM
HEK293/ET_BR	ET-1	15.6 nM	50 nM	Bosentan	10 μM
HEK293/Gα15/AT1	Angiotensin II	45.4 nM	200 nM	Telmisartan	10 μM
HEK293/Gα15/A1	Adenosine	0.99 μM	10 μM	DPCPX	10 μM
HEK293/PAR1	TRAP-6	0.89 μM	2 μM	Vorapaxar	10 μM
